# Mechanistic and reactional activation study of carbons destined for emerging pharmaceutical pollutant adsorption

**DOI:** 10.1007/s10661-025-13685-4

**Published:** 2025-02-10

**Authors:** Nora Samghouli, Imane Bencheikh, Karima Azoulay, Stina Jansson, Souad El Hajjaji

**Affiliations:** 1 Laboratory of Spectroscopy, Molecular, Modeling, Materials, Nanomaterials, Water and Environment, (LS3MNWE), Department of Chemistry, Faculty of Sciences, Mohammed V University in Rabat, Av IbnBattouta, B.P. 1014, 10000 Rabat, Morocco; 2 Department of Chemistry, Umeå University, SE-901 87, Umeå, Sweden

**Keywords:** Adsorption, Emerging pharmaceutical pollutants, Surface functional groups, Activated carbon, Chemical treatment, Physical treatment, Mechanism

## Abstract

In this review, several factors have been collected from previous studies on emerging pharmaceutical pollutant adsorption to explain and describe the mechanisms and determine the reactions involved: X-ray Photoelectron Spectroscopy (XPS), Fourier Transform Infrared Spectroscopy (FTIR), and the Boehm titration are the most used characterization techniques to determine activated carbons’ surface functional groups. Some studies have confirmed that the specific surface area and the pore structure are not more important than the functional groups present in the adsorbent surface to explain the amount of adsorption obtained and to describe correctly the interaction between the adsorbent-adsorbate. After the analysis of several studies, we concluded that to have good adsorption, it is necessary to choose the right treatment with the right activating agent to obtain the appropriate functions that will enhance the adsorption process. In addition, the functions that can react with the pharmaceutical pollutants are the oxygenated functions such as hydroxyl function, carboxylic function, and carbonyl function.

**Supplementary Information:**

The online version contains supplementary material available at 10.1007/s10661-025-13685-4.

## Introduction

In the last decades, micropollutants also called emerging pollutants have become a major problem, and their presence in the aquatic environment has been a concern for the world (Luo et al., 2014). These include pharmaceuticals such as antibiotics, analgesics, lipid regulators, anti-inflammatories, and x-ray contrast products; these pharmaceutical compounds are daily used in large quantities for human and animal health treatment. Population growth leads to the increased release of pharmaceutical compounds into the environment. As a result, these compounds are widely detected in treated effluent at the level of wastewater treatment plants. However, wastewater treatment plants are not designed to remove all pharmaceutical pollutants (Fent et al., 2006; Maldonado-Torres et al., 2018; Tambosi et al., 2010). Furthermore, these compounds affect the aquatic environment and cause adverse effects on human health and wildlife (Miller et al., 2018; Stackelberg et al., 2004; Zenker et al., 2014). The removal of pharmaceuticals has been studied using various methods such as photocatalytic degradation (Dolar et al., 2012), electrochemical removal (Sivodia & Sinha, 2020), chemical coagulation (Liu et al., 2015b), membrane filtration (Janssens et al., 2017), and adsorption method. Adsorption is one of the most studied techniques for the removal of pharmaceutical compounds via activated carbons prepared from agricultural waste. Moreover, the adsorption technique has proven to be able to achieve high removal efficiency of pharmaceutical pollutants in many scientific publications (Quesada et al., 2019). The adsorption technique remains an effective method with a simple procedure using abundant and inexpensive adsorbents (Bolong et al., 2009), such as zeolites (Wan et al., 2021), clays (Charaabi et al., 2019), and biochar (Abbas, 2020; Fernandes et al., 2019).

Biochar is a pyrogenic porous carbon produced by the thermal conversion of organic matter under the total or partial absence of oxygen, also called pyrolysis (Ok et al. 2018; Liu et al., 2021a). The process is usually carried out at moderate temperatures (350–700 °C) (Liu et al., 2015a). Due to the high external specific surface area, the abundance of surface functional groups, and the porous structure, biochar is considered an efficient adsorbent for the removal of organic and inorganic pollutants from water (Wang et al., 2017). For the biochar adsorption performance for a wider range of environmental applications, several physical or chemical modification techniques have been investigated to modify biochar properties (Wang & Wang, 2019). The physical activation process is carried out using an oxidizing gas (CO_2_, O_2_, H_2_O) in a temperature range between 800 and 1200 °C. This category of activation is affected by different factors, including carbonization temperature, precursor type, particle size, heating rate, gas flow, and carbonization time (Balahmar et al., 2017; Danish & Ahmad, 2018). The physical activation presents the advantages of being eco-friendly, and clean, and does not generate secondary waste. However, it has some disadvantages such as low mass yield, considerably long processing time, high activation temperature, and low specific surface area (Gao et al., 2020).

Chemical activation is a process based on the addition of chemicals such as alkalis (e.g., KOH, K_2_CO_3_, NaOH, or Na_2_CO_3_ (Sun et al., 2010; Guo et al., 2014; Moranville-Regourd & Kamali-Bernard, 2019), alkaline earth metal salts (e.g., AlCl_3_, FeCl_3_, or ZnCl_2_) (Bedia et al., 2020; Nassar et al., 2020; Wendimu et al., 2017), and some acids (e.g., H_3_PO_4_, H_2_SO_4_, or HCl) (Chen & Wu, 2004; El Farissi et al., 2020; Liu et al., 2021b) to the raw materials which are then heated in a temperature range between 450 and 900 °C (Sevilla & Mokaya, 2014) in the presence of inert gas (Yu et al., 2016). The main factors influencing chemical activation are the mixing method, the type of activating agent, the mass ratio of the activator, and the heating method (Sevilla & Mokaya, 2014). This type of activation has significant advantages such as low activation time and temperature, high specific surface area, lower energy consumption, developed microporosity, and high carbon yield (Nayak et al., 2017; Zhang & Shen, 2019); however, the major disadvantages of chemical activation are its corrosiveness and laborious washing process (Chen et al., 2012). The choice of the treatment process (physical, chemical, and physicochemical) is related to the nature of the functional groups that we want to obtain in the activated carbon (Qiao et al., 2002; Villacañas et al., 2006).

Several types of research have proven that the efficiency of the pharmaceutical pollutant’s adsorption is not always associated with surface active sites number the increase in the specific surface area (Álvarez-Torrellas et al., 2016; Sun et al., 2012a). For example, Liu et al. (2013) had found that the AC prepared by cattail fiber has a higher surface area (micropores and mesopores) and a larger total volume than AC prepared by animal hair (Liu et al., 2013); however, AC prepared by animal hair showed a higher sorption capacity for acetaminophen than AC prepared by cattail fiber. Similarly, Liu et al. (2012a) observed that the specific surface area of activated carbon prepared by phosphoric acid is considerably lower than that prepared by pyrophosphoric acid (Liu et al., 2012a); however, the trimethoprim adsorption capacity with the first support is considerably better than the latter. Zhou et al. (2018) had modified virgin activated carbon to improve the functional groups, and they observed that the acetone adsorption capacity of modified AC is greater than that of the virgin (Zhou et al., 2018). However, the specific surface area and the total pore volume of virgin activated carbon are greater than that of modified. The adsorption capacity of activated carbon depends mainly not only on its specific surface and its porous structure but also on the presence of functional groups on its surface. The properties of the functional groups of activated carbons are related to the nature of the raw material and the activation methods. Many types of functional groups are present in ACs; however, the functional groups containing oxygen and/or which present high acidity levels are generally considered the most important for pharmaceutical pollutant adsorption. The influence of each functional group of ACs typically contributes to the result of two factors: electrostatic interactions, which are indicated as a surface chemical property, and hydrophobic-hydrophobic interactions.

This review will be limited to the removal of emerging pharmaceutical pollutants by activated carbons using conventional pyrolysis, and chemical and physical treatment based on research published between 2007 and 2021. Where are going to study the effect of the several activation methods and the indirect effect of the activation agent on the pharmaceutical pollutant’s adsorption onto activated carbon. This will be carried out based on analyzing the factors affecting the mechanism of adsorption of emerging pollutants by activated carbon.

## Pharmaceutical pollutant adsorption via activated carbon

Activated carbons have been used to remove pharmaceuticals from water over the past decade. Tables S1, S2, S3, and S4 in the Supporting Information provide a summary of relevant information related to the pharmaceuticals’ adsorption studies reported in the literature.

An examination of the data presented in the tables (S1–S4, Supporting Information) reveals that adsorption studies of pharmaceuticals on activated carbon are classified based on therapeutic functions, such as antibiotics, anti-inflammatory drugs, analgesics, lipid regulators, diuretics, X-ray contrast products, and H2 blockers. Based on the works reported in the literature from 2007 to the present, 49.02% was studying the evaluation of the sorption capacity of activated carbon to eliminate antibiotics, including amoxicillin (AMX), cephalexin (CEX), ciprofloxacin (CPF), norfloxacin (NOR), tetracycline (TC), oxytetracycline (OXT), penicillin G (PCG), trimethoprim (TMP), chloramphenicol (CPL), and cefixime (CFX). Work focusing on the elimination of non-steroidal anti-inflammatory drugs NSAIDs account for 33.34% and included diclofenac (DCF), ketoprofen (KTP), ibuprofen (IBP), acetylsalicylic acid (ACA), and naproxen (NPX); the remaining 17.64% is consecrated for drugs frequently used as an adsorbent in some of the published work such as acetaminophen (paracetamol) (ACT), clofibric acid (CLA), caffeine (CAF), ranitidine (RNT), and iopamidol (IPD). All these drugs have been considered since 2011 as emerging pollutants mentioned in the NORMAN list, except cephalexin and ranitidine (https://www.norman-network.net/?q=node/235).

The majority of published adsorption studies focus on antibiotics and NSAIDs, which may be related to the greater concern of these classes. The removal of antibiotics from an aqueous medium is particularly difficult. In the case of non-steroidal anti-inflammatory drugs (NSAIDs), the concern originates from the high consumption of these over-the-counter (OTC) drugs, therefore, generating relatively high concentrations in wastewater.

## Chemical and morphological characteristics of the activated carbon

**Fig. 1:**
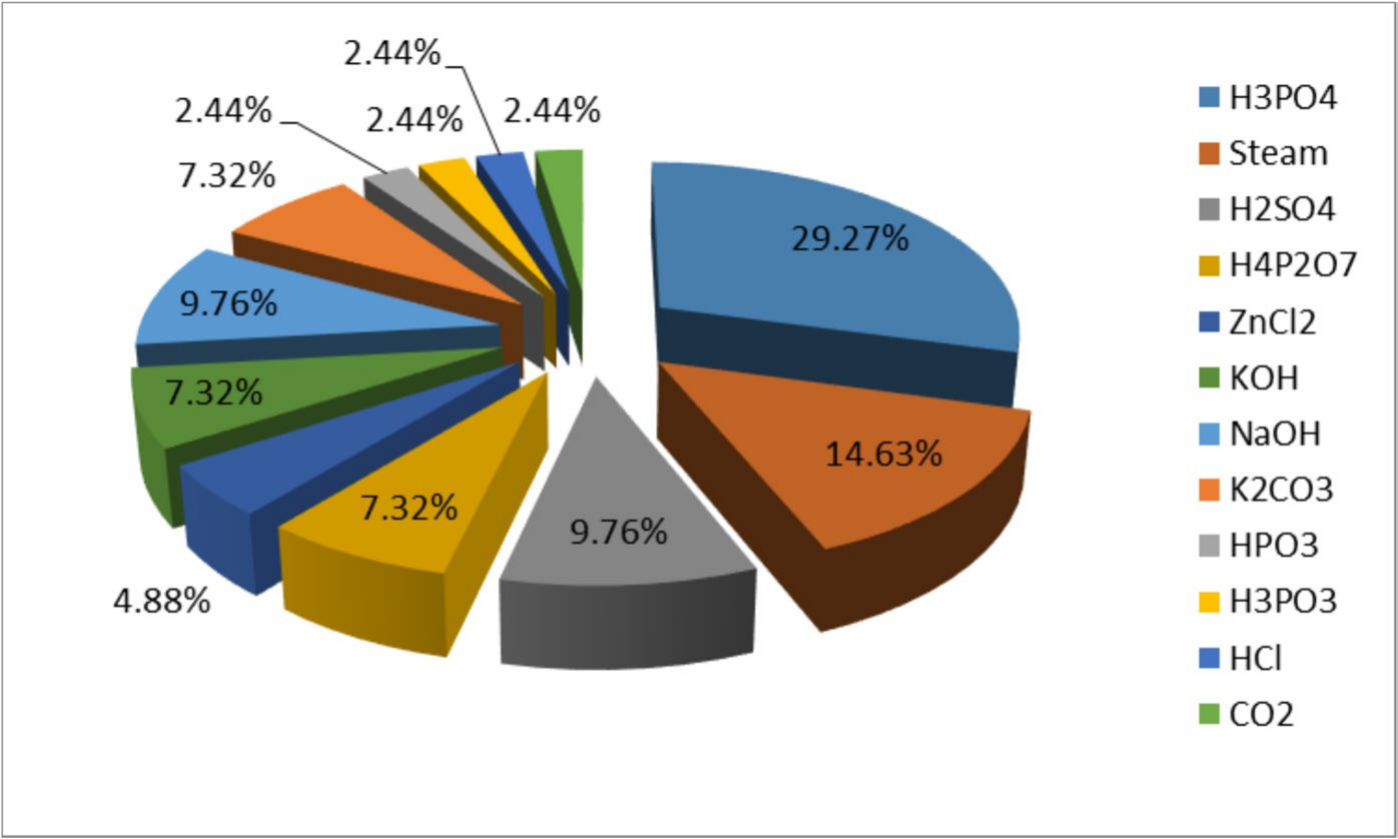
Percentage of activating agents use between 2007 and 2021

The study employed a systematic review approach, using the Scopus database as the primary information source. Inclusion and exclusion criteria focused on articles about the adsorption of emerging pharmaceutical pollutants onto activated carbons, from 2007 to 2021. A thorough search strategy, incorporating relevant keywords and time restrictions, facilitated the retrieval of pertinent publications. 29.27% of these works are concerning the chemical activation of carbons using H_3_PO_4_, 14.63% for steam physical activation, and 9.76% for H_2_SO_4_ and NaOH. H_2_SO_4_ and NaOH and 7.32% for the activation agents KOH, K_2_CO_3_, and H_4_P_2_O_7_. A few studies on activation with HPO_3_, H_3_PO_3_, CO_2_, and HCl were not detailed in the literature (Fig. 1). The wide use of phosphoric acid may be due to its availability and its environmental soundness (less toxic behavior for the environment) compared to other agents. This reagent was found to be able to improve the specific surface area and to increase the acidity and/or surface functions of the adsorbents (Girgis & El-Hendawy, 2002; Li et al., 2010).

One of the major characteristics of the adsorption process is the total surface area (the raw material per unit mass). This can influence the adsorbed quantity of activated carbon in combination with the porosity effect. In addition to the specific surface area and the material porosity of the material, the amount-adsorbed quantity is also affected by the particle size, acidity, and surface functions. These factors affect the interaction between pharmaceutical pollutants and the surface of the activated carbon (Li et al., 2002; Chang et al., 2015; Šljivić-Ivanović and Smičiklas 2020).

The specific surface area and the total pore volume increased remarkably for activated carbon prepared by KOH compared to the activated carbon prepared by NaOH and also for the activated carbon prepared by H_3_PO_4_ compared to other acids. However, the KOH activator is a special agent as other reactions occur during activation such as the formation ofK_2_CO_3_ which enters the surface to form macropores, and thus, an increase in pore size is produced. On the other hand, the production of porosity is maybe less important for all agents compared to the acid agent.

**Fig. 2:**
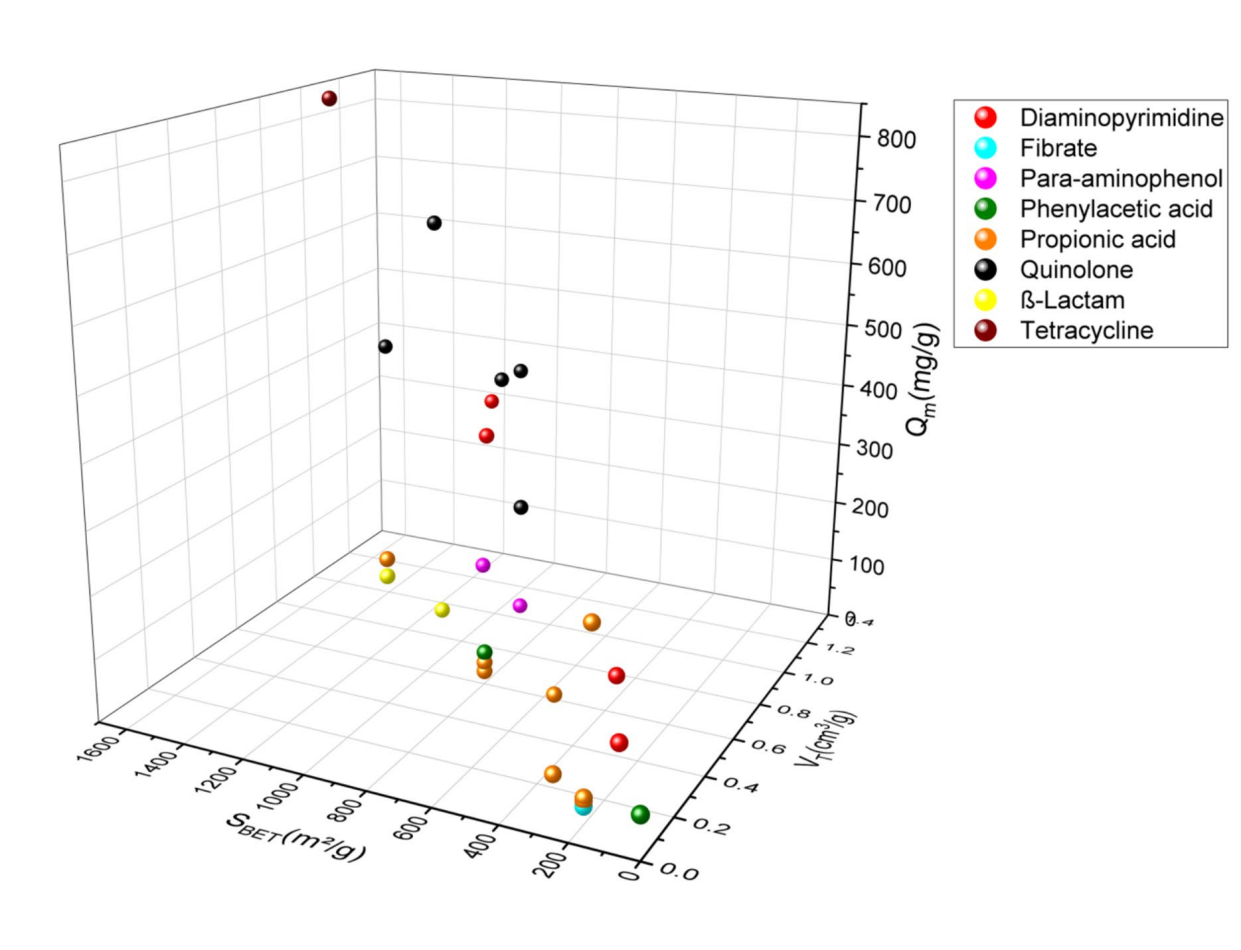
A three-dimensional (3D) plot of specific surface area, pore volume, and adsorption capacity of pharmaceutical pollutant classes on acid-activated biochar

**Fig. 3:**
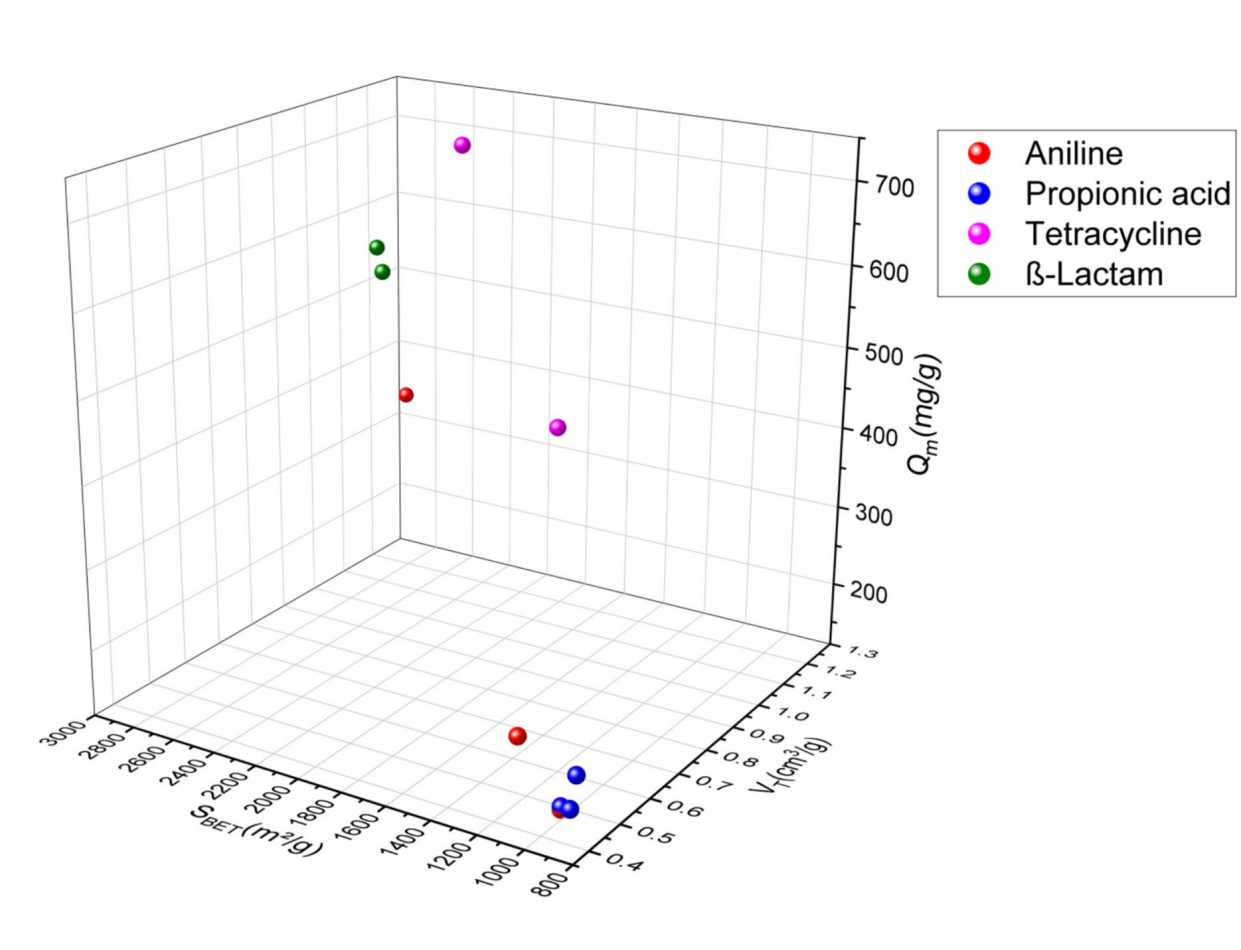
A 3D graph of specific surface area, pore volume, and adsorption capacity of pharmaceutical pollutant classes on alkaline-activated biochar

**Fig. 4:**
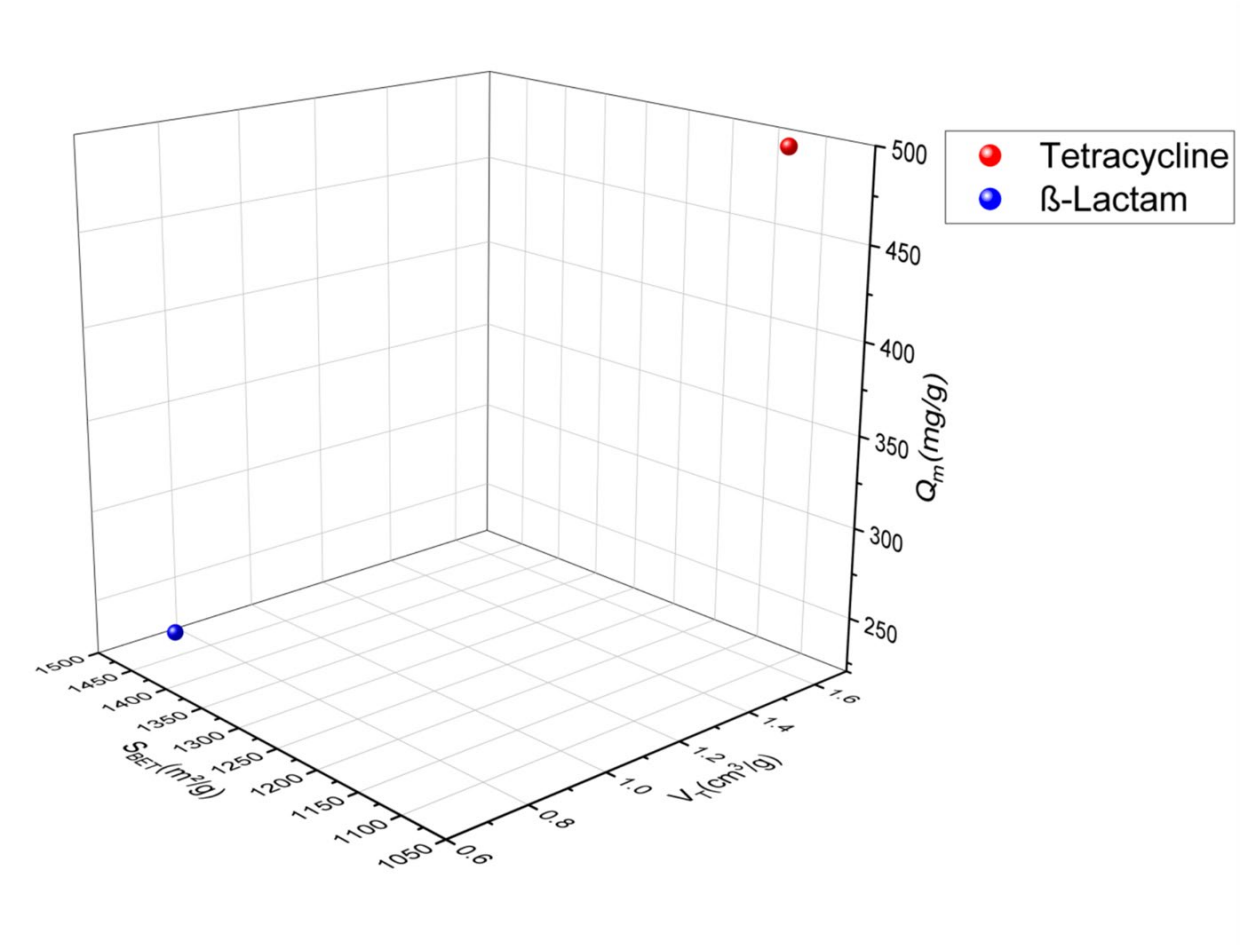
A 3D graph of specific surface area, pore volume, and adsorption capacity of pharmaceutical pollutant classes on biochar activated by neural activation agent

**Fig. 5:**
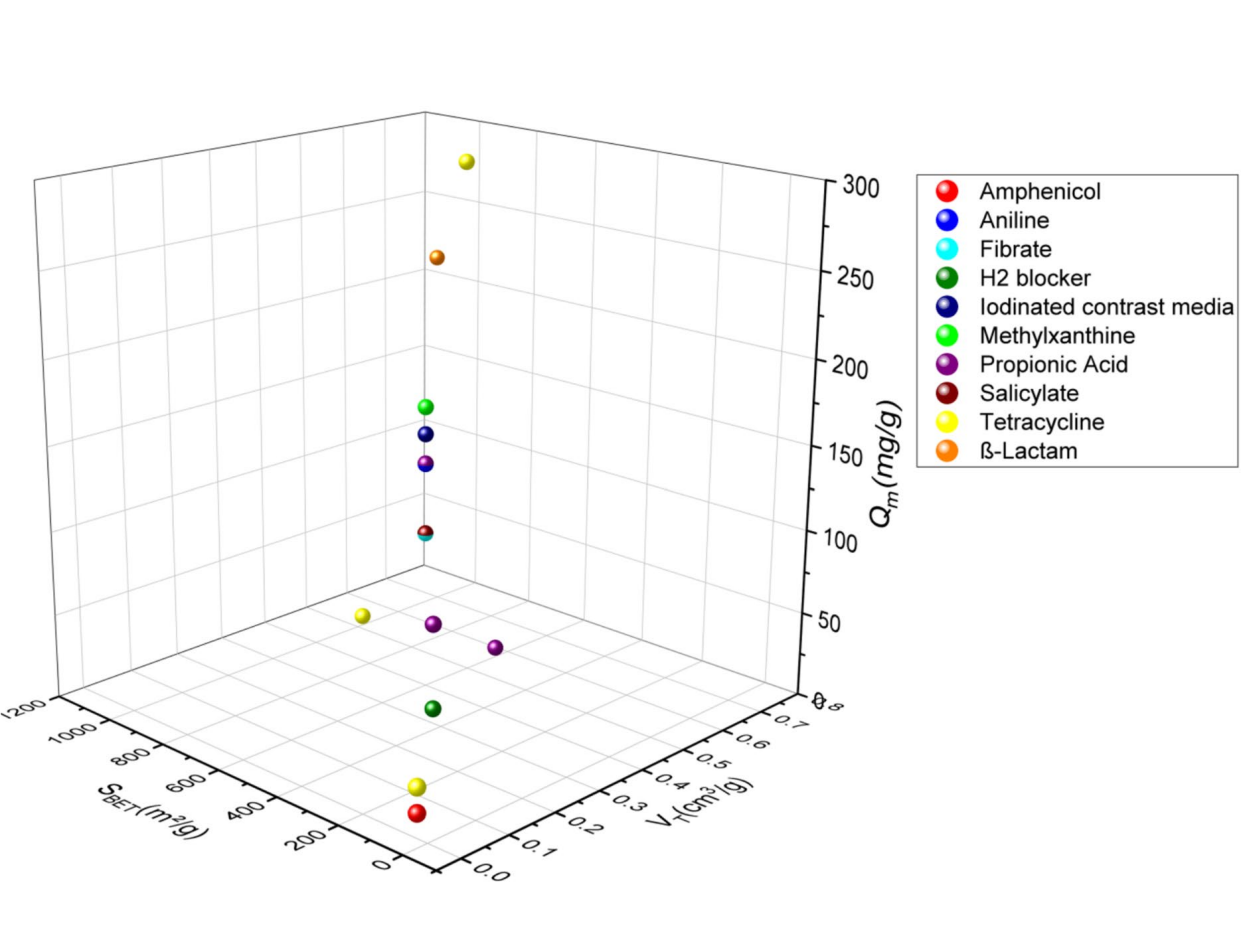
A 3D graph of specific surface area, pore volume, and adsorption capacity of pharmaceutical pollutant classes on physically activated biochar

In this review, the SSAs of activated carbons prepared from agricultural waste or any lignocellulosic material used in the adsorption of the emergent pharmaceutical pollutants via adsorption ranged from 24.4 to 1521 m^2^/g for the acid-activated materials (Fig. 2) (García-Mateos et al. 2015; Álvarez-Torrellas et al., 2016; Baccar et al., 2012; Chakraborty et al., 2018; Dubey et al., 2010, 2014; El-Shafey et al., 2012; Larous & Meniai, 2016; Liu et al., 2011, 2013; Mansouri et al., 2015; Sun et al., 2012a, 2012b; Torres-Pérez et al., 2012; Villacañas et al., 2006; Viotti et al., 2019; Xie et al., 2011), 13.39–2794 m^2^/g for alkali-activated materials (Fig. 3) (Hasanzadeh et al., 2020; Martins et al., 2015; Mestre et al., 2014; Pouretedal & Sadegh, 2014; Spessato et al., 2019; Sun et al., 2012c), 1093 m^2^/g and 1452 m^2^/g for ZnCl_2_-activated agents (Fig. 4) (Nazari et al., 2016a, 2016b; Sayğılı & Güzel, 2016), and 567.8–1055 m^2^/g (Fig. 5) (Chakraborty et al., 2018; Liao et al., 2013; Mansouri et al., 2015; Mestre et al., 2014; Mondal et al., 2016a, 2016b; Torres-Pérez et al., 2012) for physically treated biochar. Thus, the specific surface area depends strongly on the activation experimental conditions, especially on the treatment type and the used activation agents.

Porosity has an important role in the activated carbon surface. It is subdivided based on sizes and functions: micropores for high surface area and high adsorption capacity, mesopores and macropores for fast mass transport, and macropores as storage or host pores (Zhang & Shen, 2019). Based on the pore size of activated carbon, and according to the graphs, acid-activated carbons have volumes ranging from 0.002 to 1.227 cm^3^/g), for alkali-activated carbons, their pore size ranges from 0.45 to 1.191 cm^3^/g), and those activated by physical treatments, their pore sizes range from 0.024 to 0.643 cm^3^/g, and for the activated carbons by neutral agents, they have pore sizes of 0.715 and 1.569 cm^3^/g.

The amount adsorbed quantity in terms of pharmaceuticals pollutant adsorption varies from 9.744 to 845.9 mg/g for acid-activated carbons, from 1.98 to 1340.82 mg/g for alkaline-activated carbons, from 8.1 to 288.3 mg/g for physically activated carbons, and 233.1–500 mg/g for neutral-activated carbons. According to these results, the adsorbed amount of pharmaceutical pollutants using activated carbon did not show a correlation with the specific surface. However, the majority of activated carbon in this review correlated with this conclusion, in which activated carbons with a large surface area have shown a low adsorption capacity and inversely. As the AC-specific surface area did not show a correlation with the amount of adsorbed quantity, the increase of the pore diameter showed an improvement in the amount adsorbed quantity of pharmaceutical pollutants. However, adsorbents with high specific surface area and pore diameter showed a modest level of adsorption capacity. This may be due to the functionalization of the AC surface, which indicates the importance of the activation agent and the appropriate treatment choice. In addition, modification by activation can significantly increase the porosity and special biochar functional groups of the biochar, thus promoting the amount adsorbed quantity of the pharmaceutical pollutants. The adsorption capacity can be improved with higher porosity, while the functional groups determine the adsorption stability of pollutants (Zhang & Shen, 2019). Graphs 2, 3, 4, and 5 show three-dimensional (3D) graphs of the specific surface area and pore volume of activated carbon with the amount adsorbed quantity of the pharmaceutical pollutants.

## Biochar preparation

Biochar is a pyrolyzed carbon produced by the incomplete combustion of organic matter in biomass under anoxic conditions. The chemical and physical properties of the AC depend mainly on the feedstock’s nature and the pyrolysis conditions, including residence time, temperature, and heating speed (Deng et al., 2017). These parameters have an influence on the biochar formation, showing an influence on surface pH, microporous structure, specific surface area, average pore size, number, and density of functional groups (Zhao et al., 2018). Furthermore, biochar can be obtained by various pyrolysis techniques; the most commonly used are slow pyrolysis, fast pyrolysis, flash carbonization (flash pyrolysis) (Manyà, 2012), pyrolytic gasification, and microwave-assisted pyrolysis (Manyà, 2012; Ranzi et al., 2016). Biochar pyrolysis at low temperatures (about 300–450 ℃) and slow heating rates promotes the obtention of a high density of functional groups such as COOH and -OH as well as a mass yield of 35% (Deng et al., 2017; Zhao et al., 2018). Also, the increase of pyrolysis temperature by 600–700 ℃ allows the destruction of the biochar cellular structure. This allows the formation of honeycomb-shaped pores with a diameter ~ 1 µm which can be attributed to the carbon skeleton of the biological capillary structure of the lignocellulosic feedstock (Zhao et al., 2018). The carbonization (pre-treatment) step leads to a material with low porosity and fewer hydrogen and oxygen functional groups due to dehydration and deoxygenation with a highly aromatic character (Godwin et al., 2019). To develop the biochar porous structure and make to make the surface more reactive, we have to apply another treatment called activation (Zhao et al., 2018).

### Chemically activated biochar

Chemical activation can be carried out generally under inert gas (Jiménez et al., 2009) and at relatively low temperatures (between 400 and 600 °C) after impregnation of the precursor with an activating chemical agent which can be a Lewis acid (e.g., ZnCl_2_, AlCl_3_) a phosphoric acid, or sulfuric acid. Basic agents such as sodium hydroxide (NaOH) and potassium hydroxide (KOH) are also used. Oxidation of biochar with hydrogen peroxide (H_2_O_2_), potassium permanganate (KMnO_4_), ammonium persulfate ((NH_4_)2S2O8), and ozone (O_3_) has been also used to modify the surface functional groups. The chemical activation promotes a higher yield of activated carbon with a lower activation temperature, faster activation time, and better development of the pore structure (Qian et al., 2013). The most commonly used chemical biochar activation agents that have shown effective adsorption of pharmaceutical pollutants are sulfuric acid, phosphoric acid, sodium hydroxide, and potassium hydroxide.

#### Activation using phosphoric acid

Phosphoric acid can act as an acid catalyst with biochar, releasing CO_2_ at low temperatures, promoting bond cleavage reactions and the formation of cross-links via processes such as cyclisation and condensation (Chu et al., 2018; Yakout & Sharaf El-Deen, 2016), so that phosphate and polyphosphate bridges are formed which link and cross-link biopolymer fragments (Gratuito et al., 2008; Jagtoyen & Derbyshire, 1998), in addition, a small amount of CO (Chu et al., 2018; Yakout & Sharaf El-Deen, 2016). Therefore, during the reaction, the weight loss is mainly attributed to the dehydration of phosphoric acid and the degradation of some functional groups on the biochar surface.

The temperature has an important role in the chemical activation process by phosphoric acid. At high temperatures, phosphorous oxides act as Lewis acids and can form C-O-P bonds linked to the active sites of the biochar (Benaddi et al., 1998; Gratuito et al., 2008; Labruquere et al., 1997). Indeed, this temperature increase affects significantly the development of micropores (Baçaoui et al., 2001). At higher temperatures, phosphorous compounds are released from the AC surface of the activated carbon (Labruquere et al., 1997). Hence, some of the micropores formed are destroyed due to the very high oxidizing capacity of H_3_PO_4_ (Gratuito et al., 2008). The chemical activation process by H_3_PO_4_ can lead to the following reactions (Myglovets et al., 2014).

1 $$4{H}_{3}P{O}_{4}+10 C={P}_{4}+10 CO+6{H}_{2}O$$

2 $$4{H}_{3}P{O}_{4}+10 C={P}_{4}{O}_{10}+6{H}_{2}O$$

3 $${P}_{4}{O}_{10}+10 C={P}_{4}+10 CO$$

These reactions were also reported by Shi et al. (2019) who have used plants containing phytic acid as biomass precursors considered rich in phosphoric acid (Shi et al., 2019).

#### Activation using sulfuric acid

Sulfuric acid is a strong oxidant; it allows the sulfonation of the carbon structure of the aromatic rings (Kastner et al., 2012; Lu & Love, 2005; Toda et al., 2005) which is accompanied by partial oxidation of the cellulosic group (-CH_2_-OH). This results from a considerable increase in the surface area and pore structure of biochars (Kastner et al., 2012; Xiong et al., 2018). Chemical activation by sulfuric acid is influenced by temperature. Therefore, sulfur generated in the elemental form at relatively low temperatures causes a blockage at the pore’s level (Guo et al., 2005, p. 4).

However, at high temperatures, the elemental sulfur is vaporized, which leads to the development of pores on the biochar surface (Guo et al., 2005, p. 4). During activation, dehydration of H_2_SO_4_ can generate internal pores by gasifying and burning the precursor carbon content of the precursor (Guo et al., 2005, p. 4). When sulfuric acid is in excess, the pores expand via a conversion process that converts micropores into mesopores and macropores. The formation reactions of H_2_O, CO, and H_2_ can be represented as follows (Guo et al., 2005, p. 4)(Cotton et al., 1988):

4 $$\left[{C}_{n}{H}_{x}{O}_{y}\right]+{H}_{2}S{O}_{4}\to {H}_{2}O\uparrow +S \left(element\right)+\left[{C}_{n}{H}_{x}{O}_{y}+3\right]$$

5 $${H}_{2}O+\left[{C}_{n}{H}_{x}{O}_{y}\right]\to {H}_{2}\uparrow +CO\uparrow +\left[{C}_{n}-1{H}_{x}{O}_{y}\right]$$

6 $${C}_{n}{H}_{2n}{O}_{n}+{H}_{2}S{O}_{4}\to nC+{H}_{2}S{O}_{4}\cdot n{H}_{2}O$$

#### Activation using hydrochloric acid

Chemical treatment of biochar with HCl can increase the oxygenated groups on the surface, such as phenol groups, ether groups, and lactone groups. The possible reaction that leads to the formation of a pyrone structure is the following:
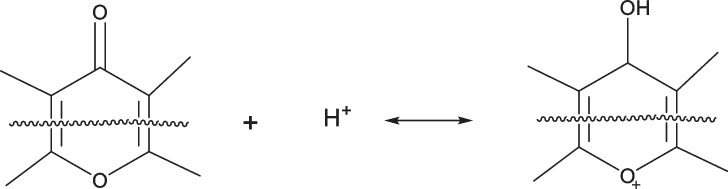


Activation by HCl can increase the oxygen content in the structure by 10%, which may be due to the chemisorption of water molecules by delocalized π-electrons in the graphite carbon.

$${C}_{\pi }+{H}_{3}{O}^{+}\leftrightarrow {C}_{\pi }-{H}_{3}{O}^{+}$$

Cπ is the graphite carbon surface plate with the maximum number of π-electrons (Chen & Wu, 2004; Leon y Leon et al., 1992).

#### Activation mechanism of biochar by phosphoric and sulfuric acid—cellulose

To understand the biochar activation mechanism by either H_2_SO_4_ or H_3_PO_4_ we have proposed detailed mechanisms for the reaction of these agents on the main or major components present in the biochar generated via lignocellulosic precursor thermal activation (cellulose, lignin, and graphite).

**Fig. 6:**
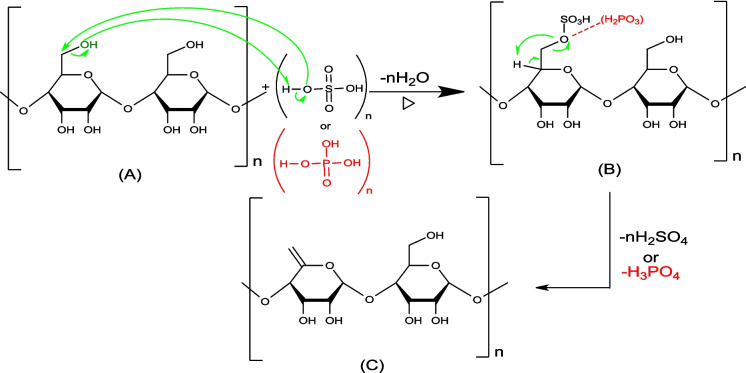
Mechanism of primary cellulose alcohols: attack by phosphoric or sulfuric acid

**Fig. 7:**
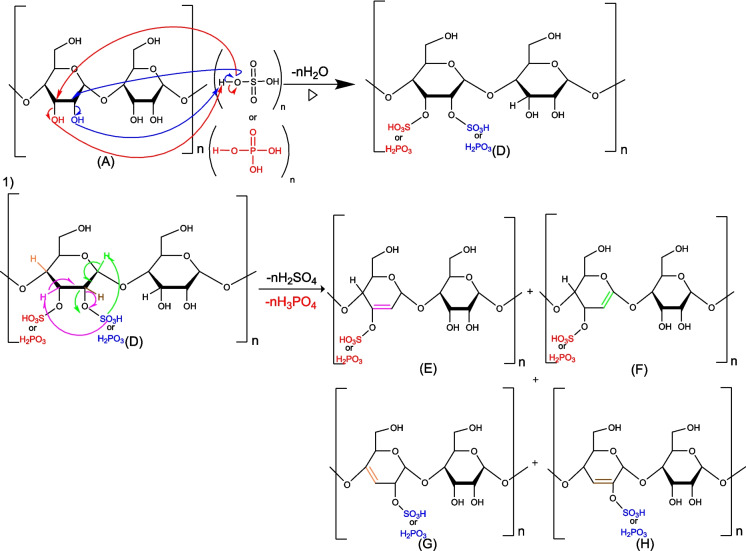
Mechanism of secondary alcohols of cellulose: attack by phosphoric or sulfuric acid

The primary or secondary alcohols in cellulose can be dehydrated with sulfuric or phosphoric acid (molecules B and D) to produce alkene (molecules C, E, F, G, and H) (Figs. 6 and 7).

The primary alcohol mechanism (Fig. 6) is E2 elimination, which can occur in 2 steps: the first one is the alcohol protonation, and the second step is a proton elimination in one step. For the secondary alcohol mechanism (Fig. 7), intramolecular dehydration takes place according to an E1 mechanism.

The temperature of the reaction must be closely monitored. At high temperatures, dehydration occurs as expected.

**Fig. 8:**
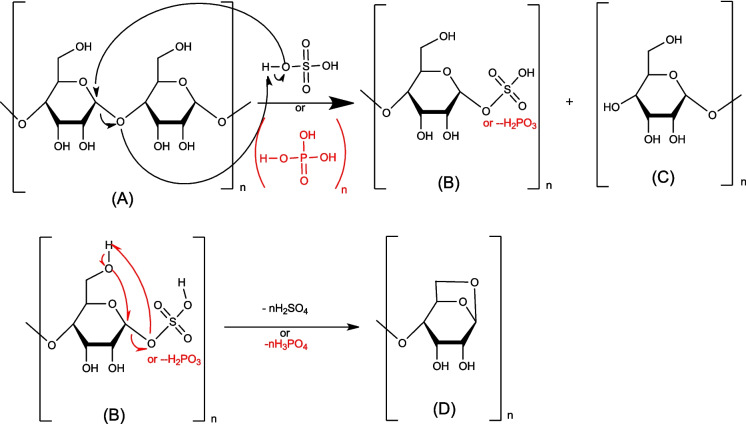
Mechanism of cellulose depolymerization: attack by phosphoric or sulfuric acid

As shown in the following mechanism, in the first step of the cellulose activation, the reaction mainly involves depolymerization via the cleavage of the bond between glucose molecules leading to the production of B and C. In addition, the release of sulfuric acid (-H_2_SO_4_) from molecule B can undergo a cyclisation reaction to obtain molecule D (see Fig. 8).

#### Activation mechanism of biochar by phosphoric and sulfuric acid—lignin

**Fig. 9:**
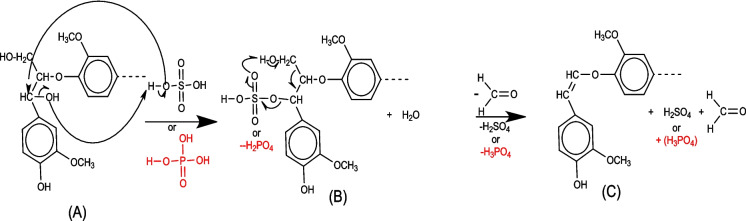
Lignin mechanism: attack by phosphoric or sulfuric acid

The lignin primary alcohols can lead in the first step to protonation of the alcohol with the formation of the aldehyde (B) and in the second step the mechanism can lead to an alkene formation (C) with a release of aldehyde (- H_2_C = O) (Fig. 9).

#### Activation mechanism of biochar by phosphoric and sulfuric acid—graphite

**Fig. 10:**
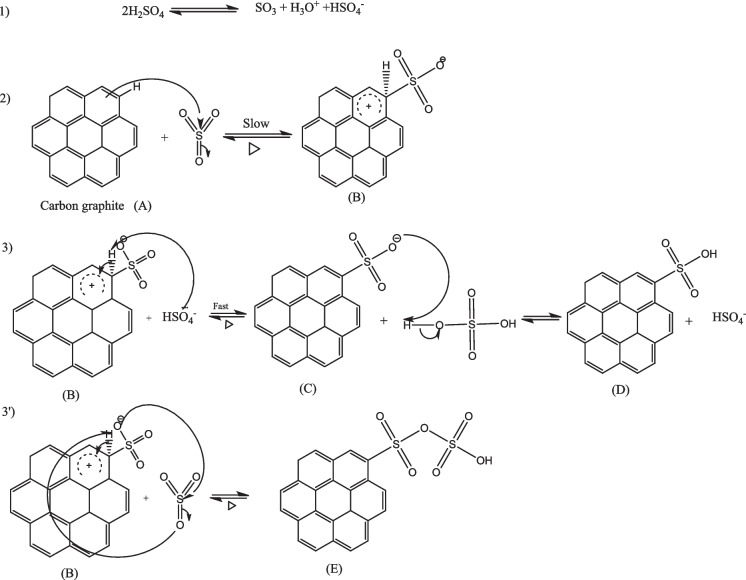
Mechanism of graphite sulfonation: attack by sulfuric acid

The graphite sulfonation mechanism is described in four steps. In the first step and according to reaction number 1, we have the formation of sulfur anhydrides (SO_3_), H^+^, and HSO4- ions from the activation agent H2SO4. In the second step, SO_3_ reacts with graphite and generates a positively charged and delocalized graphite-cyclohexadienyl cation, also called the Wheland intermediate or arene complex (B). In the third step, HSO^4−^ attacks the proton of the arenium ion (B) and forms a graphite-sulphonic acid anion (C). At the same step, we may have anion protonation (C), which generates a neutral graphite-sulphonic acid molecule. For the 3′ mechanism, we supposed that the anion (C) reacts with SO_3_ to generate a polysulfate bridge (E) on graphite (Fig. 10).

#### Biochar activation mechanism of biochar using hydrochloric acid—cellulose

**Fig. 11:**
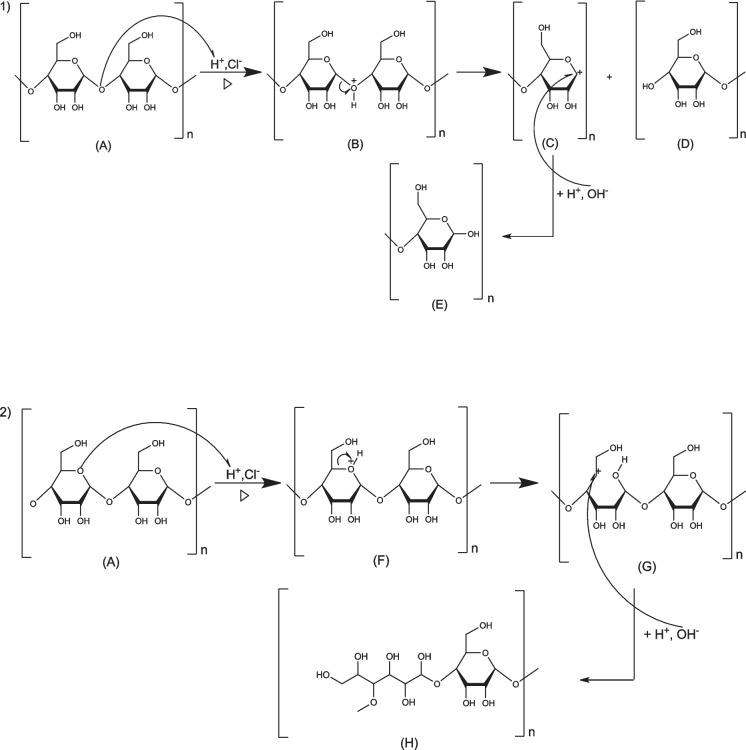
Mechanism of hydrochloric acid activation of cellulose

**Fig. 12:**
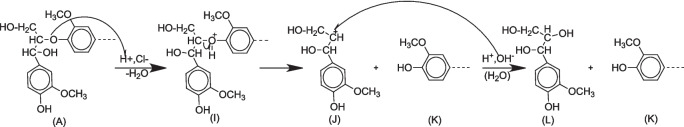
Mechanism of hydrochloric acid activation of lignin

Cellulose and lignin activation using HCl is achieved by a first-order nucleophilic substitution mechanism (Sn1). First, a proton of the acid interacts with the C–O–C bond and polarizes the carbons of the acetal bond (B, F, and I). Then, the C-O is cleaved by an Sn1 mechanism, and the conjugated acid is converted to a carbonium ion (C, G, and J), then releasing glucose (D and E) for reaction 1, cellulose and a compound (H) for reaction 2, and cellulose plus compound (K) for lignin. A nucleophilic attack for the lignin compound (J) leads to the formation of the compound (L) (see Figs. 11 and 12).

#### Biochar activation mechanism of biochar using hydrochloric acid—lignin

### Basic activation of biochars

Based on the literature, chemical activation by the dehydrating agents “alkaline hydroxides such as KOH or NaOH” develops porosity by the formation of lacunae, thus increasing the specific surface area. Sodium (Na) and potassium (K) play an elementary role in the chemical activation process by partial gasification, which allows their diffusion into the carbon surface internal structure, leading to an enlargement of the existing pores and the development of porosity. Since the potassium radius is larger than the sodium radius, potassium should cause a much greater widening of the intermediate layers and thus produce more micropores compared to sodium. Activation by these agents leads to C–C and C–O–C hydrochar bonds breaking. In addition to the redox reactions that reduce MOH to alkali metal M and H_2_. These reductions lead to the release of Na and K, which then oxidize the carbon to sodium carbonate (M_2_CO_3_) which in turn leads to further secondary reactions that form the gases CO_2_ and CO. The mechanisms of water–gas formation and the formation of alkali-containing constituents are as follows (Ahmed & Theydan, 2012; Boujibar et al., 2019; Chiu & Lin, 2019; Hunsom & Autthanit, 2013; Islam et al., 2017; Norouzi et al., 2018; Paredes-Laverde et al., 2019):

Where M: K or Na.

7 $$4MOH+(-C{H}_{2})\to {M}_{2}C{O}_{3}+{M}_{2}O+{H}_{2}$$

8 $$6MOH+2C\to 2{M}_{2}C{O}_{3}+2M+3{H}_{2}$$

9 $${M}_{2}C{O}_{3}\to C{O}_{2}+{M}_{2}O$$

10 $$4MOH+C\to 4M+C{O}_{2}+2{H}_{2}O$$

11 $${M}_{2}O+C\to 2M+CO$$

12 $${M}_{2}C{O}_{3}+2C\to 2M+3CO$$

#### Activation mechanism by NaOH and KOH

**Fig. 13:**
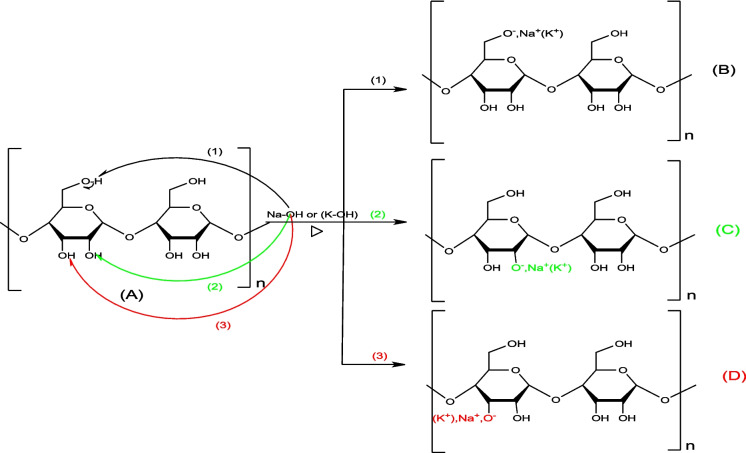
Mechanism of NaOH/KOH activation of cellulose

**Fig. 14:**
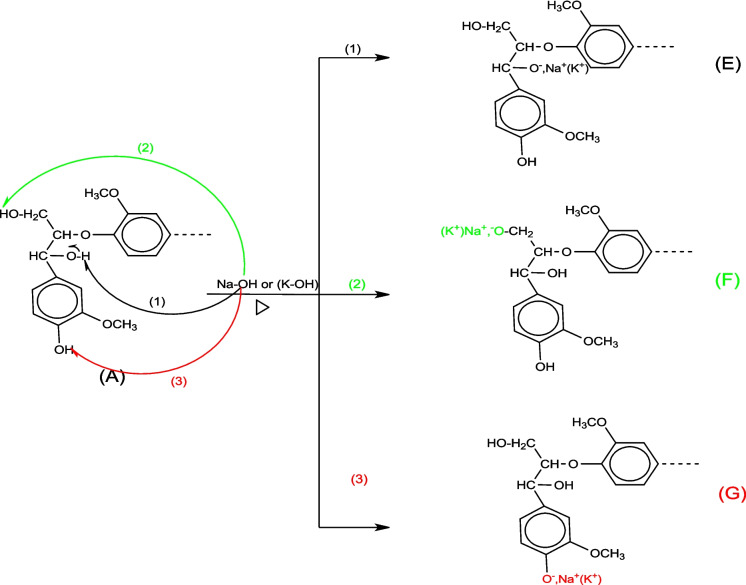
Mechanism of NaOH and KOH activation of lignin

The biomass basic treatment (NaOH/KOH), called alkali treatment or mercerization, leads to alkoxide ions (B, C, D, E, F, and G) resulting from the ionization of biochar hydroxyl groups. The mechanisms of NaOH/KOH with the -OH groups of cellulose and lignin are presented in Figs. 13 and 14, respectively.

### Activation by Lewis acid

ZnCl_2_ is a Lewis acid, which means that it can interact with the unbound electrons of the oxygen atoms (OH groups) in the precursor (Khadiran et al., 2014), leading to the porosity increase (Khadiran et al., 2014; Wang & Liu, 2017). Activation with ZnCl_2_ occurred in a temperature range between 300 and 600 °C (Delgado & Mendez, 2011). It is considered a milder agent that can be suitable for making porous carbonaceous materials (Wang & Liu, 2017). As the temperature increases a large amount of ZnCl_2_ volatilizes with a decrease in pore size and specific surface area (Delgado & Mendez, 2011). The absence of unrelated oxygen molecules in the precursor reduces the interaction capacity of ZnCl_2_ with the precursor (Khadiran et al., 2014). The high acidity of ZnCl_2_ facilitates the degradation of the lignin and cellulose structure during impregnation (Delgado & Mendez, 2011; Khadiran et al., 2014). The precursor functional groups react with ZnCl_2_ to form Zn–O complexes, which are then followed by water removal. Subsequently, the biopolymers are transformed into randomly oriented carbon structures, which produce cavities (Delgado & Mendez, 2011; Khadiran et al., 2014), leading to a specific surface area increase (Delgado & Mendez, 2011). During chemical activation, ZnCl_2_ acts as a dehydrating agent, promoting carbon condensation reactions, while inhibiting tar formation and carbon gasification, resulting in high carbon yields. This indicates the high yield of carbon (Wang & Liu, 2017).

#### Biochar activation mechanism using ZnCl2

**Fig. 15:**
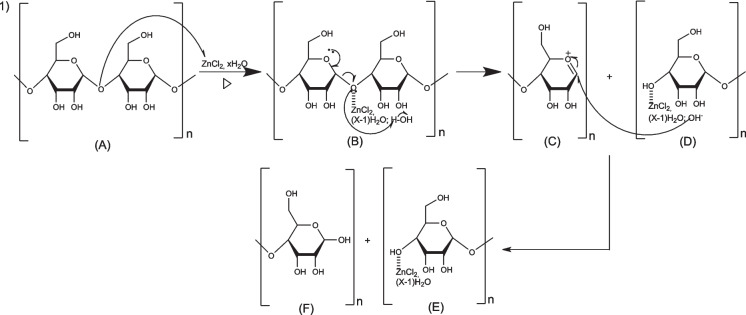
Mechanism of cellulose activation by ZnCl_2_

The proposed mechanism for activation by ZnCl_2_ on cellulose is shown in Fig. 15. In the first step, we have the coordination of ZnCl_2_ to the glycosidic oxygen, which acts as a Lewis acid, leading to the breaking of the glycosidic bond and the formation of the compound (B). In the second step, we have the hydrolysis of cellulose to glucose in the presence of H_2_O molecules of hydrated ZnCl_2_, which act as a nucleophile.

### Physical activation

Physical activation is a carbon activation at high temperatures, generally 800–900 °C (Ahmad et al., 2012), and in the presence of steam or carbon dioxide in the form of oxidizing gases (Arami-Niya et al., 2011). These gaseous activation agents can partially erode the carbon atoms of the biochar matrix via the gasification reactions C-H_2_O and/or C-CO_2_ according to the following endothermic reactions:

13 $$C+C{O}_{2}\to 2CO$$

14 $$C+{H}_{2}O\to CO+{H}_{2}$$

The specific surface area of the biochar can be significantly enhanced whereby reactive carbons in the carbonized material can be selectively removed during the thermal activation process (Ahmad et al., 2012), and the pores enclosed in the biochar matrix can be opened and interpenetrated with other pores. This allows the development of the microporous structure as well as a small contribution from the mesoporous ones (Cao et al., 2017). In general, the specific surface area, porosity, and distribution of activated carbons vary considerably depending on the biomass type, the activation gas, and the reaction conditions (Mansouri et al., 2015). The use of carbon dioxide as an oxidizing agent favors the development of microporosity, whereas water vapor favors a porosity with larger dimensions (Mestre et al., 2014).

### Surface functional groups of activated biochar

The biochar properties and the chemical and physical activation mechanisms of biomass are closely related to the functional groups on the surface, mainly the oxygen-containing groups including hydroxyl, carboxyl, carbonyl, and phenolic groups. These surface functions may exist in the biomass or may be formed after pyrolysis during the production of virgin biochar, either in the chemical or physical activation step. Combining the information from the previous chapters and Tables S1, S2, S3, and S4 in Supporting Information, Fig. 16 shows a schema of different surface functions of activated biochar.

**Fig. 16:**
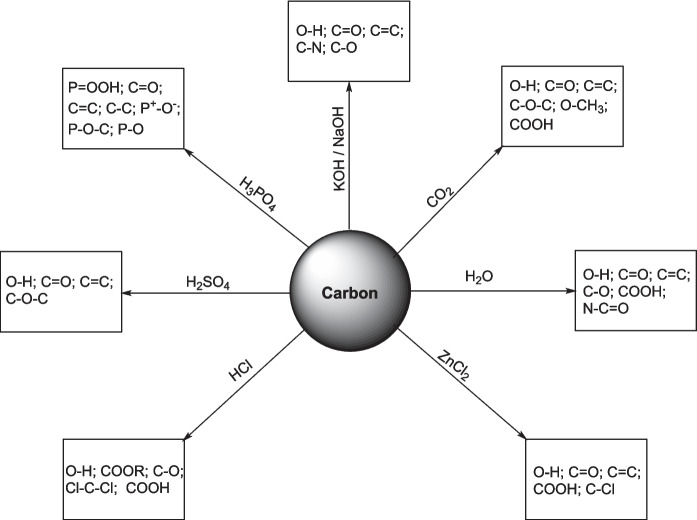
Schema of different surface functional groups of activated carbon using different agents

## Applications of activated biochar on the adsorption of emerging pharmaceutical pollutants

### Acidic activation of biochar

The activation of the lignocellulosic precursors by acidic agents such as H_3_PO_4_, H_4_P_2_O_7_, HPO_3_, H_2_SO_4_, and HCl allows us to obtain a material with several surface functional groups such as hydroxyl, carboxyl phenol, ester, lactone, quinone, ketone, aromatic rings, amide, alcohols, nitrile, ether, and aromatic amine aldehydes following infrared spectroscopy (FTIR) and Boehm titration. This was reported by Sun et al. (2012a), which revealed a difference between the biochar activation using H_3_PO_4_ and H_4_P_2_O_7_. H_4_P_2_O_7_ had shown a higher acidity compared to H_3_PO_4_, therefore a significant amount of oxygenated functional groups on the AC surface (Sun et al., 2012a). Also, Larous and Meniai (2016) have illustrated that the impregnation of biochar with H_2_SO_4_ increased in the acid groups (Larous & Meniai, 2016).

At the reactional level, the main mechanisms that can occur between pharmaceutical pollutants and the activated carbons functional groups are electrostatic interactions (cation-π bonding), electron-donor-π-acceptor interactions (EDA), hydrogen bonds, Lewis’s acid–base interactions, and cation exchange. This was reported by Álvarez-Torrellas et al. (2016), who studied the effect of phosphoric acid activation of the peach pit (PP) and rice husk (RH) on the removal of ibuprofen and tetracycline and their adsorption mechanism (Álvarez-Torrellas et al., 2016). They revealed that the adsorption capacity of tetracycline is higher than that of ibuprofen; this was explained by their ring contents which exist at the levels of these drugs, as ibuprofen contains a single ring while tetracycline has four. These high adsorption capacities of these pollutants were attributed to the π-π EDA interactions related to the graphitic structure with the rings of these drugs. Therefore, the existence of ketone and nitro functional groups in the tetracycline molecule leads to a stronger attraction to carbon surfaces. The mechanism of adsorption of these drugs by phosphoric acid-activated carbons proved that the hydroxyl groups allow the removal of tetracycline through π- π EDA interactions. Thus, the carboxylic, ester, and lactone groups of AC-PS react with the functional groups of tetracycline as well as forming hydrogen bonds. Álvarez-Torrellas et al. (2016) proved that carboxylic, ketone, ionic radical structures, lactone, ester, and quinone groups were involved in the removal of ibuprofen and tetracycline (Álvarez-Torrellas et al., 2016). Also, Liu et al. (2012a) revealed a possible reaction between the removal of trimethoprim by activated carbons prepared using H_3_PO_4_, H_4_P_2_O_7_, H_3_PO_3_, and HPO_3_ such as hydrogen bonding, cation-π bonding, π-π electron-donor–acceptor interaction (EDA), and Lewis’s acid–base interaction (Liu et al., 2012a). The methoxy groups (-OCH_3_) that occur on the benzene ring of the TMP molecule are electron-donating moieties that enrich the benzene ring with electrons. This allows them to interact strongly with the electron-depleted carbon surfaces via π-π EDA interactions. At pH = 6, all of the TMP was protonated which facilitates a strong interaction between the -NH^3+^cation and the π of the graphitized carbon. Another hypothesis was proposed by Liu et al. (2012a), according to which hydrogen bonds and Lewis’s acid–base interactions have occurred between the -NH^3+^ in TMP and the oxygen groups at the carbon surface (Liu et al., 2012a). However, we also found that the properties of activated carbon are significantly influenced not only by the functional groups of the activated carbon surface. Also, the specific surface area, porosity, surface charge, and morphological characteristics of the activated carbon may have a great influence on the explanation of the pharmaceutical pollutants’ adsorption mechanisms.

### Alkaline activating agent of biochar

Generally, the activation of biochar using alkalis allows for obtaining oxygenated groups such as hydroxyl, carboxyl, aromatic rings, aldehydes, and amine functions according to infrared spectroscopy (FTIR), XPS analysis, and Boehm titration. These functional groups allow electrostatic interactions between activated carbon and pharmaceutical pollutants. This was shown by Hasanzadeh et al. (2020), who proved the efficiency of prepared activated carbons in the removal of CFX molecules due to the presence of hydroxyl (-OH) and carboxylic (-COOH) groups, which are characterized by their engagement in hydrogen bonding (Hasanzadeh et al., 2020). Also, Liu et al. (2012b) cited that for TC adsorption, the surface of the alkali-activated biochar has more oxygen functional groups, which facilitates the formation of hydrogen bonds with the TC zwitterionic form (Liu et al., 2012b). Furthermore, Martins et al. (2015), indicated in the case of tetracycline removal that the mechanism can contribute to the adsorption through the formation of hydrogen bonds since the zwitterionic form is predominant (Martins et al., 2015), whereas the case of the study of paracetamol removal (Spessato et al., 2019) showed that the paracetamol amount adsorbed quantity is strongly influenced by the biochar high porous structure (Spessato et al., 2019). Also, Spessato et al. (2019) indicate that the previously mentioned interactions can be better explained by the molecular orbital theory which is used to determine the most likely functional groups that are involved to describe the adsorption mechanism based on the pHpzc, hence the speciation of the adsorbate functional groups can be modified by changing the pH of the solution, as the pharmaceutical pollutant adsorption rate is altered with the change in pH. Moreover, according to the boundary molecular orbitals, adsorption can be explained by electron donor–acceptor interactions (EDA) (π-π hydrophobic EDA and n-π EDA), hydrogen bonding interaction, and π-hydrogen bonding (hydrogen bonding-aromatic nuclei). However, adsorption studies of pharmaceutical pollutants by KOH and NaOH-activated biochars have shown that oxygen functional groups could be beneficial for the organic pollutant’s adsorption. For the removal of pharmaceutical pollutants by KOH- and NaOH-activated carbons, the authors have not investigated the reactions and bonds involved in the adsorption process on K_2_CO_3_-activated carbons, which may indicate that the adsorption can be attributed to the mechanisms of KOH- and NaOH-activated carbons since they contain the same surface functions.

The majority of scientific papers did not explain in detail the reaction mechanisms of pharmaceutical pollutants’ adsorption using carbons activated by physical and alkaline agents.

## Conclusion

The pharmaceutical industry is one of the most productive sectors in recent years, due to the development of the world pharmaceutical economy because of the increased consumption of pharmaceuticals associated with the growing prevalence of chronic diseases, aging populations, and clinical evolution. This increase in consumption has caused environmental pollution and destruction. Pharmaceutical residues, now considered as emerging contaminants, are being discharged into water and soil, thus affecting human beings. Wide ranges of low-cost biosorbent materials are becoming increasingly common, while these wastes contain a porous structure and contain oxygen groups that can better adsorb these emerging pharmaceutical pollutants. Therefore, these precursors are modified by physical or chemical treatments to develop and increase oxygenated groups such as hydroxyl groups, carboxyls, and ketones. This review aimed to analyze the adsorption mechanism of emerging pharmaceutical pollutants and activated carbon and to suggest activation mechanisms and the role of the activator in biomass modification.

## Supplementary Information

Below is the link to the electronic supplementary material.

Supplementary file1 (DOCX 103 KB)

## Data Availability

No datasets were generated or analysed during the current study.
