# Supplementary material for: Mechanistic and reactional activation study of carbons destined for emerging pharmaceutical pollutant adsorption
Source: Environ Monit Assess. 2025 Feb 10;197(3):259. doi: 10.1007/s10661-025-13685-4 (PMC11811452; doi:10.1007/s10661-025-13685-4)
Supplement: Supplementary file 1 — Supplementary file1 (DOCX 103 KB) [file 10661_2025_13685_MOESM1_ESM.docx]

**Supporting information to:**

**Mechanistic and reactional activation study of carbons destined for emerging pharmaceutical pollutants adsorption**

Samghouli Nora^a^, Bencheikh Imane^a^, Azoulay Karima^a^, Jansson Stina*^b^*^,*^, and El Hajjaji Souad^a^

*^a^Laboratory of Spectroscopy, Molecular, Modelling, Materials, Nanomaterials, Water and Environment, (LS3MNWE), Department of Chemistry, Faculty of Sciences, Mohammed V University in Rabat, Av Ibn Battouta, B.P. 1014, Rabat 10000, Morocco*

*^b^ Department of Chemistry, Umeå University, SE-901 87 Umeå, Sweden*

*Corresponding Author: [stina.jansson@umu.se](mailto:stina.jansson@umu.se)

**Tables S1-S4, 11 pages.**

The tables contain summarized data from other studies and are used as a source to support the scheme in Figure 16.

**Table S1**. Parameters used to prepare activated carbon by acids to remove pharmaceutical emerging pollutants from water. Data on the activated biochar producing using acids, experimental conditions used in the syntheses of these activated carbons, their surface specific, the total volume of pores, surface functional groups, and the adsorption conditions (adsorbed quantities, and optimal adsorption conditions) of the elimination of emerging pharmaceutical pollutants will be provided. Antibiotics: amoxicillin (AMX), cephalexin (CEX), ciprofloxacin (CPF), norfloxacin (NOR), tetracycline (TC), oxytetracycline (OXT), penicillin G (PCG), trimethoprim (TMP), chloramphenicol (CPL), and cefixime (CFX). Pharmaceuticals and other substances: acetylsalicylic acid (ACA), acetaminophen (paracetamol) (ACT), caffeine (CAF), clofibric acid (CLA), diclofenac (DCF), ibuprofen (IBP), iopamidol (IPD). ketoprofen (KTP), naproxen (NPX), and ranitidine (RNT).

| Biomass feedstock  (as stated in reference) | Activator | Activation Temperature (°C) | Impregnation ratio | Surface functional group(s) | S_BET_ (m^2^∙g^-1^) | V_tot_ (cm^3^∙g^-1^) | Adsorbates | Adsorption Capacity (mg∙g^-1^) | Adsorption condition | Reference |
| --- | --- | --- | --- | --- | --- | --- | --- | --- | --- | --- |
| Cyperus alternifolius | H_3_PO _4_ | 450 | 1:2 | Hydroxyl group (-OH); Alkyl groups (-CH_n_); Ester groups; Carboxylic groups; Lactone; Phenol | 1066 | 1.151 | CPF | 377.4 | C=200-1000 mg/L; pH=5.05;  T=50°C ; t=10 h ; Dose=1g/L | (Sun et al. 2012a) |
| Olive stones | H_3_PO _4_ | 550 | 1:3 | - | 990 | 0.91 | ACT | 108.3 | C=1-20 mg/L; Solution’s pH T=15°C ; t=n/a; Dose=0.1g/L ; | (García-Mateos et al. 2015) |
| Olive-waste cakes | H_3_PO _4_ | 450 | 2:3.5 | - | 793 | 0.59 | IBP | 12.6 | C=10.04-19.78 mg/L; pH=2.01 ;  T=25°C ; t=26h  Dose=1.5g/L ; | (Baccar et al. 2012) |
|  |  |  |  |  |  |  | KTP | 24.7 |  |  |
|  |  |  |  |  |  |  | NPX | 39.5 |  |  |
|  |  |  |  |  |  |  | DCF | 56.2 |  |  |
| Trapanatans husk | H_3_PO _4_ | 470 | 1:2.3 | Carboxylic, phenolic, and lactonic groups | 1274 | 1.087 | NOR | 638.7 | C=25.5–159.7 mg/L; pH=7.6;  T=21±1°C ; t=25h Dose=0.07 g/L | (Xie et al. 2011) |
| Olive stones | H_3_PO _4_ | 450 | 1:3 | - | 1106 | 0.560 | AMX | 158.8 | C=5-100 mg/L ; pH=4.3 ;  T=25 C ; t=24h Dose=0.3g/L | (Mansouri et al. 2015) |
|  |  |  |  |  |  |  | IBP | 178.0 |  |  |
| Peach stones | H_3_PO _4_ | 400 | - | Hydroxyl groups; Ketone group; Carboxylic groups; Esters and lactones. | 1521 | 0.90 | TC | 845.9 | C=2-80 mg/;  pH=n/a;  T=30 °C; t=72 h; Dose=2.4 g/L | (Álvarez-Torrellas et al. 2016) |
| Rice husk |  |  |  | –OH and/or C-O in the carboxylate group or ethers; Acid groups; Ketone; Carboxylic acid; Ester; Lactone and quinone. | 278 | 0.26 | IBP | 239.8 |  |  |
| Cattail fiber | H_3_PO _4_ | 500 | 1 :2.5 | C=C bond in benzene derivatives; Carbonyl groups: C-O bond and C-C bond. | 907 | 1,00 | NOR | 197.0 | C=12.8–127.8 mg/L; pH=5; T=25 °C; t=50 h; Dose=0.15g/L | (Liu et al. 2013) |
|  |  |  |  |  |  |  | ACT | 23.4 | C=6-60.5 mg/L;  pH=5; T=25 °C; t=9 h; Dose=0.6 g/L |  |
| Sugarcane bagasse | H_3_PO _4_ | 400 | 1:2 | Amide groups; Alkyl groups; Aromatic cycle; C-N bond. | 557 | 0,583 | IBP | 13.5 | C=1–50 mg/L ; pH=2 ;  T=15 °C; t=12 h ; Dose=1.66 g/L | (Chakraborty et al. 2018) |
| Lotus stalk | H_3_PO _4_ | 500 | 1:2.5 | Function -C = O belongs to carboxyl groups; COO− function; Asymmetric stretch of P-O-C and symmetrical vibration of P-O; C-C and C-O vibrations in esters, ether, or phenol | 1114 | 1.17 | TMP | 333 | C=29–87 mg/L ;  pH=6; T=25 °C; t=3days  Dose=0.2 g/L | (Liu et al. 2012a) |
| Lotus stalk | H_3_PO _4_ | 450 | 1:2 | C–C and C–O vibrations in acids, alcohols, phenols, ethers, and esters; carboxylic groups; C=O stretches in aromatic rings; O–H stretch for carboxyl and phenol functional groups or adsorbed water and hydrogen bonding; P–O vibration in a chain of P–O–P | 1031.840 | 0.7398 | CEX | 66.2 | C=4–16 mg/L; pH=2.5; T=20 °C; t=32.5 h | (Liu et al. 2011) |
| Lotus stalk | H_4_P_2_O_7_ | 500 | 1:2.5 | Function -C = O belongs to carboxyl groups; COO− function; Asymmetric stretch of P-O-C and symmetrical vibration of P-O; C-C and C-O vibrations in esters, ether or phenol. | 940 | 0.845 | TMP | 345 | C=29–87 mg/L;  pH=6;  T = 25 °C; t=3 days;  Dose=0.2 g/L | (Liu et al. 2012a) |
| Cyperus alternifolius | H_4_P_2_O_7_ | 450 | 1:1 | Hydroxyl group (OH); Alkyl groups (CHn); Ester groups; Carboxylic groups. Lactone.  Phenol. | 1040.3 | 1.227 | CPF | 381.7 | C=200–1000 mg/L; pH=5.05; T=30 °C; t=10 h; Dose=1g/L | (Sun et al. 2012a) |
| Arundo donax | H_4_P_2_O_7_ | 600 | 1:0.75 | Hydroxyl group O-H;  Elongation of C-H; aromatic C = C bond; carboxylic acid or phosphate ester C – O group. | 1463 | 1.09 | CPF | 418.4 | C=400-1200 mg/L; pH=4.71;  T=20 °C; t=36 h; Dose=1 g/L | (Sun et al. 2012b) |
| Artemisia vulgaris | H_2_SO_4_ | 450 | 20 avec 30 ml à 60% (v / v) | Hydroxy group, H-bonded OH stretch, and amide N–H stretch peak  ; Aromatic C–H stretching, C=C ring stretch, C=C aromatic ring stretch, C–H bending; Methyl C–H asymmetric and symmetric; Amide C=O stretch and Amide N–H bending; C–N stretch; C–O stretch. | 358.20 | - | IBP | 16.9 | C=10–50 mg/L;  pH 2–4; T=25 °C; t=5 h; Dose=2 g/L | (Dubey et al. 2010) |
| Olive stones | H_2_SO_4_ | 550 | 1:1 | Nitrile group (C≡N); Carboxylic acids; Ether groups; Aromatic amine. | 83.72 | - | DIC | 11.0 | C=25-150 mg/L;  pH= 4.2; T=23 ± 2°C; t=3 h; Dose =5 g/L | (Larous and Meniai 2016) |
| Date palm leaves | H_2_SO_4_ | 160 | - | O–H stretching vibrations (Hydrogen bond);  C–H stretching vibrations (CH_2_);  Group –C=O corresponds to (lactone and carboxylic acids);  Asymmetric and symmetrical –COO^-^ group (carboxylate) or aromatic ring C=C; C–O bond of hydroxyl groups and ether structures. | 24.4 | - | CPF | 125.0 | C=50-300 mg/L;  pH=6; T=45 °C; t=48 h; Dose=2 g/L | (El-Shafey et al. 2012) |
| Raspberry leaves | H_2_SO_4_ | 450 | - | Phenolics; Aliphatic hydrocarbons; Conjugated diene ;  Phenyl ring substitution band; Aromatic rings; Ketones ;  Aldehyde; Amines. | - | - | IBP | 22.7 | C=10-20mg/L; pH=3-5; T=30±1 °C; t=5 h; Dose=1.75 mg/L | (Dubey et al. 2014) |
|  |  |  |  |  |  |  | NPX | 17.7 |  |  |
|  |  |  |  |  |  |  | CLA | 9.7 |  |  |
| Lotus stalk | HPO_3_ | 500 | 1:2.5 | Function -C = O belongs to carboxyl groups; COO− function; Asymmetric stretch of P-O-C and symmetrical vibration of P-O. | 279 | 0.418 | TMP | 119 | C=29–87 mg/L;  pH=6; T = 25 °C; t= 3 days;  Dose=0.2 g/L | (Liu et al. 2012a) |
|  | H_3_PO_3_ |  |  | Function –C = O belongs to carboxyl groups; COO− function; Asymmetric stretch of P-O-C and symmetrical vibration of P-O; Aromatic substitution. | 125 | 0.123 |  | 118 |  |  |
| Moringa oleifera | HCl | 25 | 1:3 | O-H vibration stretching; Aliphatic chain (C-H) stretching vibrations carbonyl group stretching vibrations; [aromatic groups](https://www.sciencedirect.com/topics/engineering/aromatic-group); Presence of lignin; C-O stretching of alcohols and phenols | 4.4 | 0.002 | DCF | 66.2 | C=20–200 mg/g;  pH = 7; T = 45 °C; t=6 h; Dose =0.1g/L | (Viotti et al. 2019) |

**Table S2**. Parameters used to prepare activated carbon by Lewis acids to remove pharmaceutical emerging pollutants from water. Data on the activated biochar producing using Lewis acids, experimental conditions used in the syntheses of these activated carbons, their surface specific, the total volume of pores, surface functional groups, and the adsorption conditions (adsorbed quantities, and optimal adsorption conditions) of the elimination of emerging pharmaceutical pollutants will be provided. Antibiotics: amoxicillin (AMX), cephalexin (CEX), ciprofloxacin (CPF), norfloxacin (NOR), tetracycline (TC), oxytetracycline (OXT), penicillin G (PCG), trimethoprim (TMP), chloramphenicol (CPL), and cefixime (CFX). Pharmaceuticals and other substances: acetylsalicylic acid (ACA), acetaminophen (paracetamol) (ACT), caffeine (CAF), clofibric acid (CLA), diclofenac (DCF), ibuprofen (IBP), iopamidol (IPD). ketoprofen (KTP), naproxen (NPX), and ranitidine (RNT).

| Biomass feedstock  (as stated in reference) | Activator | Activation Temperature (°C) | Impregnation ratio | Surface functional group(s) | S_BET_ (m^2^∙g^-1^) | V_tot_ (cm^3^∙g^-1^) | Adsorbates | Adsorption Capacity (mg∙g^-1^) | Adsorption condition | Reference |
| --- | --- | --- | --- | --- | --- | --- | --- | --- | --- | --- |
| Walnut shell | ZnCl_2_ | 450 | 1:1 | Carboxylic acid; Phenol; Carbonyl, Lactone. | 1452 | 0.715 | CEX | 233.1 | C=50-150 mg/L;  pH = 6.5;  T= 30 °C; t=350 min; Dose=0.48 g/L | (Nazari et al. 2016b) |
|  |  |  |  | O–H out-of-plane bending; -C–H bending and C–Cl stretch; –C–H out-of-plane bending; –C–O stretch (strong); –N–O stretch; C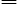C stretching; –C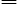O stretch (strong) amide; –C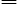O stretch and stretch; –C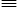C stretching; –CH stretch ; –OH stretch, H-bonded; –NH2 stretching. |  |  |  |  | C=100-200 mg/g; pH=6,5 ;  T=30°C;  Dose=0.6 g/L | (Nazari et al. 2016a) |
| Lycopersicon esculentum | ZnCl_2_ | 600 | 1:6 | OH group; C = C bond of the aromatic ring; C-O bond of the phenol group. | 1093 | 1.569 | TC | 500 | C=200-800 mg/L; pH=5.7;  T=35°C ; t=5 h; Dose=200 mg/L | (Sayğılı and Güzel 2016) |

**Table S3**. Parameters used to prepare activated carbon by alkaline activation agent to remove pharmaceutical emerging pollutants from water. Data on the activated biochar producing using alkaline activation agent, experimental conditions used in the syntheses of these activated carbons, their surface specific, the total volume of pores, surface functional groups, and the adsorption conditions (adsorbed quantities, and optimal adsorption conditions) of the elimination of emerging pharmaceutical pollutants will be provided. Antibiotics: amoxicillin (AMX), cephalexin (CEX), ciprofloxacin (CPF), norfloxacin (NOR), tetracycline (TC), oxytetracycline (OXT), penicillin G (PCG), trimethoprim (TMP), chloramphenicol (CPL), and cefixime (CFX). Pharmaceuticals and other substances: acetylsalicylic acid (ACA), acetaminophen (paracetamol) (ACT), caffeine (CAF), clofibric acid (CLA), diclofenac (DCF), ibuprofen (IBP), iopamidol (IPD). ketoprofen (KTP), naproxen (NPX), and ranitidine (RNT).

| Biomass feedstock  (as stated in reference) | Activator | Activation Temperature (°C) | Impregnation ratio | Surface functional group(s) | S_BET_ (m^2^∙g^-1^) | V_tot_ (cm^3^∙g^-1^) | Adsorbates | Adsorption Capacity (mg∙g^-1^) | Adsorption condition | Reference |
| --- | --- | --- | --- | --- | --- | --- | --- | --- | --- | --- |
| Cork | KOH | 800 | 1:1 | Carboxylic acids;  Cyclic ether; phenols | 948 | 0.47 | IBP | 174.4 | C=20-150 mg/L; pH=5;  T= 30°C ; t=6 h Dose= 0.67 g/L | (Mestre et al. 2014) |
| Hymenaea Courbaril .L | KOH | 500 | 1:3 | O-H vibration; stretching vibration C = O; stretching vibrations C = C;  stretching vibrations of C-O and C-N | 2794 | -- | ACT | 356.3 | C= 25-500 mg /L; pH=5;  T= 30 °C; t=4 h;  Dose= 1 g/L | (Spessato et al. 2019) |
| Date press cake | KOH | 700 | 1:3 | O-H vibration; C - O stretch vibration; C-N-C stretching C -C vibrations;  C-H stretching vibrations | 2760 | 1.0752 | CFX | 571.5 | C=50-800mg/L ; pH=4;  T= 22°C; t= 4 h; Dose= 1 g/L; | (Hasanzadeh et al. 2020) |
|  | NaOH |  |  | O-H vibration; stretching vibration C = O; C = C stretching C-N-C vibrations; C-O stretch vibrations | 2623.2 | 1.0057 |  | 557.9 |  |  |
| Cotton linters | NaOH | 550 | 1:3 | C = C double bond; Carbonyl function C = O; Single carbon-oxygen bond of C-O phenol; -CH aromatic rings | 2143 | 0.948 | OXT | 1340.8 | C= 208.2-530.3 mg/L;  pH=3.10–3.36 ;  T=50°C; t=24 h; Dose= 1 g/L. | (Sun et al. 2012c) |
| Macadamia nut shells | NaOH | 500 | 1:3 | O-H groups; C=O in carboxyl, alkenes, and aromatic rings; C-C bond; C–O bond in carboxylic acids, alcohols, phenols, and esters; Aromatic cycle. | 1524 | 0.826 | TC | 455.8 | C =250-800 mg/L; pH=3; T=25°C; t=2h;  Dose=1 g/L | (Martins et al. 2015) |
| Vinewood | NaOH | 600 |  | C=O groups; Bond C=C;  Hydroxyl groups; C-CH_3_ bond;  C-O bond. | 13.397 | --- | AMX | 2.69 | C=20- 200 mg/L; pH=2;  T=45°C; t=8 h;  Dose=0.4 g/L | (Pouretedal and Sadegh 2014) |
|  |  |  |  |  |  |  | TC | 1.98 |  |  |
|  |  |  |  |  |  |  | PCG | 8.41 |  |  |
|  |  |  |  |  |  |  | CEX | 7.08 |  |  |

**Table S4**. Parameters used to prepare activated carbon by physical activation to remove pharmaceutical emerging pollutants from water. Data on the activated biochar producing using physical activation, experimental conditions used in the syntheses of these activated carbons, their surface specific, the total volume of pores, surface functional groups, and the adsorption conditions (adsorbed quantities, and optimal adsorption conditions) of the elimination of emerging pharmaceutical pollutants will be provided. Antibiotics: amoxicillin (AMX), cephalexin (CEX), ciprofloxacin (CPF), norfloxacin (NOR), tetracycline (TC), oxytetracycline (OXT), penicillin G (PCG), trimethoprim (TMP), chloramphenicol (CPL), and cefixime (CFX). Pharmaceuticals and other substances: acetylsalicylic acid (ACA), acetaminophen (paracetamol) (ACT), caffeine (CAF), clofibric acid (CLA), diclofenac (DCF), ibuprofen (IBP), iopamidol (IPD). ketoprofen (KTP), naproxen (NPX), and ranitidine (RNT).

| Biomass feedstock  (as stated in reference) | Activator | Activation Temperature (°C) | Surface functional group(s) | S_BET_ (m^2^∙g^-1^) | V_tot_ (cm^3^∙g^-1^) | Adsorbates | Adsorption Capacity (mg∙g^-1^) | Adsorption condition | Reference |
| --- | --- | --- | --- | --- | --- | --- | --- | --- | --- |
| Cork powder | Steam | 800 | Carboxylic acids; Cyclic ether; Phenol groups. | 750 | 0.50 | IBP | 119.0 | C=20-150 mg/L; pH=5;  T= 30°C; t=6 h; Dose= 0.67 g/L | (Mestre et al. 2014) |
|  |  |  |  |  |  | ACT | 118.6 |  |  |
|  |  |  |  |  |  | CAF | 153.4 |  |  |
|  |  |  |  |  |  | IPD | 136.9 |  |  |
|  |  |  |  |  |  | CLA | 75.8 |  |  |
|  |  |  |  |  |  | ACA | 75.9 |  |  |
| sugar beet pulp |  | 850 | - | 821 | 0.6430 | TC | 288.3 | C=50-300 mg/L; pH=3.6; T=n/a °C; t= 5 days; Dose=50 mg/L | (Torres-Pérez et al. 2012) |
| peanut hulls |  |  |  | 829 | 0.4028 |  | 28.0 |  |  |
| Bamboo charcoal |  | 700 | C-C bond; Group C-O; C=N bond; Function C=O; COO^-^ function. | 67.80 | 0.024 | TC | 22.7 | C=20-80 mg/L; pH=7.0; T=30°C; t=24 h; Dose=1g/L | (Liao et al. 2013) |
|  |  |  |  |  |  | CPL | 8.1 |  |  |
| Mungbean pod |  | 650 | C-H bond; C-N bond; Nitro group; N-H bond; C-O bond. | 405 | 0.2853 | RNT | 12 | C=100-200 mg/L; pH=2;  T=28°C; t=200 min; Dose=5.25 g/L | (Mondal et al. 2016a) |
|  |  |  |  |  |  | IBP | 62.5 | C=5-100 mg/L; pH=2;  T=20 C; t=120 min; Dose=0.1 g/L | (Mondal et al. 2016b) |
| Sugarcane bagasse |  | 500 | Amide; Alcohol; Alkyl; Aromatic cycle; C-O bond. | 557 | 0.583 | IBP | 11.9 | C=1–50 mg/L; pH=2; T=20 °C; t=18 h; Dose=2.33 g/L | (Chakraborty et al. 2018) |
| Olive stones | CO_2_ | 800 | - | 1055 | 0.733 | AMX | 216.9 | C=5-100 mg/L; pH=4.3; T=25°C; t=24 h; Dose=0.3 g/L | (Mansouri et al. 2015) |
|  |  |  |  |  |  | IBP | 388.0 |  |  |
